# Effect of a Home-Based Virtual Reality Intervention for Children with Cerebral Palsy Using Super Pop VR Evaluation Metrics: A Feasibility Study

**DOI:** 10.1155/2015/812348

**Published:** 2015-09-17

**Authors:** Yuping Chen, Sergio Garcia-Vergara, Ayanna M. Howard

**Affiliations:** ^1^Department of Physical Therapy, Georgia State University, Atlanta, GA 30302-4019, USA; ^2^School of Electrical and Computer Engineering, Georgia Institute of Technology, Atlanta, GA 30332, USA

## Abstract

*Objective. *The purpose of this pilot study was to determine whether Super Pop VR, a low-cost virtual reality (VR) system, was a feasible system for documenting improvement in children with cerebral palsy (CP) and whether a home-based VR intervention was effective. *Methods. *Three children with CP participated in this study and received an 8-week VR intervention (30 minutes × 5 sessions/week) using the commercial EyeToy Play VR system. Reaching kinematics measured by Super Pop VR and two fine motor tools (Bruininks-Oseretsky Test of Motor Proficiency second edition, BOT-2, and Pediatric Motor Activity Log, PMAL) were tested before, mid, and after intervention. *Results.* All children successfully completed the evaluations using the Super Pop VR system at home where 85% of the reaches collected were used to compute reaching kinematics, which is compatible with literature using expensive motion analysis systems. Only the child with hemiplegic CP and more impaired arm function improved the reaching kinematics and functional use of the affected hand after intervention. *Conclusion.* Super Pop VR proved to be a feasible evaluation tool in children with CP.

## 1. Introduction

Cerebral palsy (CP), the leading cause of childhood physical disability, profoundly affects physical function in children [1]. About 1 in 303 school-aged children in the US is diagnosed with CP and about half of these children have impaired arm function or difficulty in reaching, grasping, and manipulating objects [2–5]. Current interventions for children with CP emphasize the importance of repeated practice of functional activities in various contexts with provisions for performance feedback [6–13]. However, these children are often reluctant to engage in repeated practice of activities due to boredom of the training or movement limitations of the children [14].

Virtual reality (VR) systems offer a way to create an interactive, motivating environment for intensive training practice for children with CP [15, 16]: these systems can provide immediate feedback as players see themselves immersed in the virtual world and can observe themselves as they move. Researchers have begun to investigate the use of VR in helping children with CP improve their arm function [17–23]. A recent meta-analysis of literature on the use of VR to treat arm function in children with CP reported that home-based interventions had better intervention effects than clinic-based interventions [24]. Studies that used reaching kinematics measured by motion analysis systems showed larger effect sizes than studies which used standardized clinical assessment tools [24], as reaching kinematics may have captured the children's best capacity. While reaching kinematics by motion analysis systems is a reliable and sensitive measurement, it is expensive, and access to it is limited since most of these motion analysis systems lack portability (e.g., Vicon system or Qualisys system) and are typically located in a hospital or laboratory environment. Also, when children need to travel to an unfamiliar setting, such as a clinic or laboratory, for testing, their movement patterns may change such that their typical performance is not reflected (e.g., laboratory gait) [25, 26]. This affects the accuracy of measurements using a motion analysis system. Thus, while reaching kinematics are sensitive enough to detect changes, the environment in which they are collected has limited access, lacks portability, and introduces unfamiliarity to the child.

**Figure 1:**
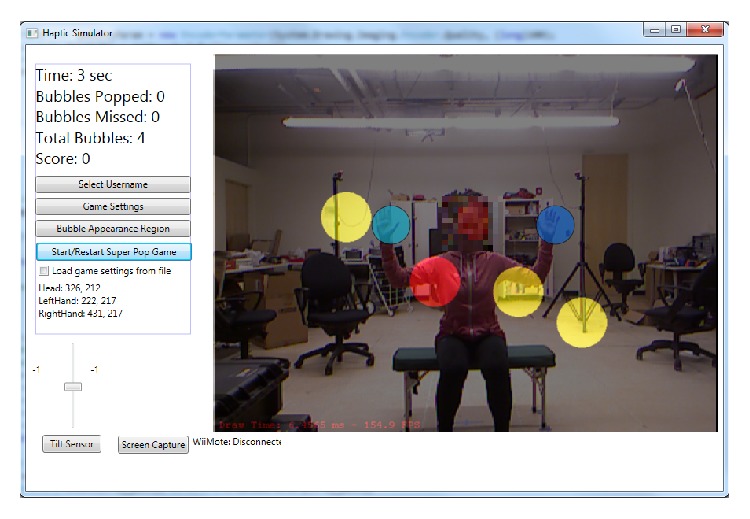
Super Pop VR game. The player needs to pop the yellow bubbles but to avoid the red bubble. The two blue bubbles represent the player's hands. Please note that the locations, sizes, shapes, appearing time, and retaining time of the bubbles can be adjusted.

**Figure 2:**
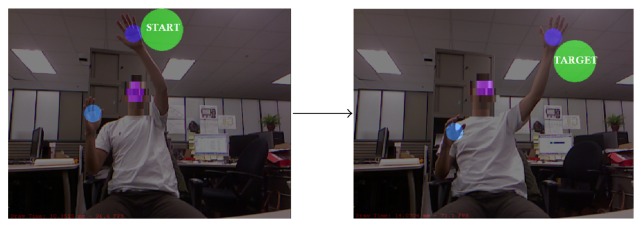
Super Pop VR game with “Super Bubble.” The green bubble on the screen is a “Super Bubble.” The player was moving his left hand from Super Bubble 1 to Super Bubble 2. The sizes of “Super Bubble” can be adjusted.

We recently developed a virtual reality evaluation game called Super Pop VR using a low-cost VR system to increase access to a motion analysis system at home [27, 28]. Since Super Pop VR is designed as an interactive game, the person involved in the test is also not aware of being evaluated. The VR system consists of a laptop running a 64-bit Windows operation system, a 3D depth camera (Microsoft Kinect) to capture and store the 3D coordinates of the user's arm movements, and software called Super Pop VR. The player is immersed in a virtual world where virtual bubbles appear on the screen surrounding the player (Figure 1). The goal is for the player to pop as many bubbles as possible in a certain amount of time by moving his or her arms. The locations, sizes, shapes, appearing time, and retaining time of the bubbles can be adjusted. We can also set up virtual obstacles (e.g., “red circles”) that participants need to avoid during playing to prevent score deduction. This can be used to train the participants' selection control strategies. Within the game, there is a set of green bubbles called “Super Bubbles” that can be used to measure the player's arm trajectory. The locations of these Super Bubbles can be easily adjusted using the game interface (Figure 2). For example, if the evaluation is designed to measure changed range of motion (ROM), the location of the Super Bubbles can be set to be spaced at a greater angle than the player's common ROM. When the player pops these bubbles, the 3D Kinect captures and saves the user's upper-body 3D joint coordinates of the assessment, to be run through a metrics code to compute the spatiotemporal reaching kinematics of the user in real time (e.g., movement time, path length, shoulder range of motion, elbow range of motion, and number of movement units). Detailed algorithms of the metrics code have been published elsewhere [27, 28]. Children with different levels of physical disabilities can use Super Pop VR, such as CP, traumatic brain injury, stroke, or even orthopedic conditions. However, the children will need to have sufficient cognitive ability to understand that the images inside the computer monitor are corresponding to their own movements.

In this study, we pilot-tested three children with CP using the Super Pop VR system to evaluate their reaching kinematics at home, in order to assess the treatment effect after an 8-week home-based VR intervention. Eleven healthy children with typical development were also tested using the Super Pop VR system to serve as the comparison “norm” group. The purpose of this study was to determine (1) whether Super Pop VR was a feasible system for documenting the improvement changes in function in children with CP and (2) to replicate and expand our previous finding to determine whether an 8-week home-based VR intervention was effective in improving children's arm function using Super Pop VR and standardized assessment tools to document the improvement in reaching kinematics, fine motor function, and active use in the more affected hand while the child was in their natural environment. We specifically found two children with an uncommon diagnosis of hypotonic CP to see whether VR intervention would be of benefit to these children having no obvious impairment in arm function.

## 2. Methods

### 2.1. Participants

Three children with CP (3 girls, mean age = 9 ± 1.73 years) and 11 children with typical development (6 girls and 5 boys, mean age = 8.87 ± 1.87 years) participated in the study (see Table 1). Children with CP were recruited from an outpatient physical therapy clinic or referred by a pediatric neurologist in the Metro Atlanta area, and children with typical development were recruited by word of mouth, also in the Metro Atlanta area.

Children with CP were included if they (1) were able to follow verbal instructions during the evaluation, (2) were able to reach forward for more than half of their arm length, and (3) had normal or corrected-to-normal vision and hearing. We specifically set our inclusion criteria very broad as our feasibility study was interested in examining whether our measurement matrices worked across a wide spectrum of children with CP. Children with CP were excluded if they (1) had received or were scheduled to receive surgery or botulinum toxin type A injections in the arms within the preceding 6 months or during the planned study period, (2) had severe attention deficit, or (3) had seizures which could be triggered by TV light. All these criteria were confirmed by the child's therapist or pediatric neurologist. All children with typical development were in general good health without any known neurological or orthopedic diagnoses, as confirmed by their parents or legal guardians.

### 2.2. VR Intervention

All children with CP received an 8-week VR intervention using the commercial EyeToy Play VR system, which consisted of a USB camera, a television, EyeToy Play and EyeToy Play 2 software, and PlayStation 2. In this system, the camera was used as a capturing and tracking device to place the children within the VR environment so that they could interact with virtual objects or events. Ten games were selected from the EyeToy Play and EyeToy Play 2 software, including Wishi Washi, Soccer Craze, Slap Stream, Kung Foo, Rocket Rumble, Beat Freak, Bubble Pop, DIY, Goal Attack, and Table Tennis [29]. Children were seated with proper trunk support and were encouraged to reach as quickly as possible toward the virtual objects (e.g., rats, animated enemies, and rockets) that appeared in any direction on the screen. The selected games were targeted to train children with CP to reach outwards and upwards and sometimes to reach with both arms. All the games allowed children to perform goal-directed reaching in all directions, to practice anticipatory reaching movements with moving targets and to experience satisfaction with easy achievement. The games were played at the easy level as they were challenging enough for these children with CP. Each week, children were asked to play 30-minute all-direction games, 40-minute reaching upwards games, 40-minute reaching outwards, and 40-minute bimanual reaching games. Based on game content, children might use one or two arms to play the games during training; however, only the more affected arm was evaluated. The more affected arm was determined and confirmed by their physical therapist and parents.

The children with CP were required to play 30 minutes per day, 5 days per week, for 8 weeks. The parents were asked to fill in a game-play log to record the playing time and the games the children played in each session. The children were also required to maintain their regular physical and occupational therapy sessions throughout the intervention period.

### 2.3. Measurements

For children with CP, three types of measurements were used: reaching kinematics using the Super Pop VR game, the fine motor scale of the Bruininks-Oseretsky Test of Motor Proficiency second edition (BOT-2), and the Pediatric Motor Activity Log (PMAL) [30]. Children were evaluated three times: before intervention, mid intervention (after completing 4 weeks of the VR intervention), and immediately after intervention (within 1 week after completing the 8-week VR intervention). We specifically added a midpoint evaluation to examine whether a 4-week intervention would be sufficient for children with CP. Children with typical development also played the Super Pop VR game at their home once and their data served as the “norm” comparison.

*Reaching Kinematics*. Four 75-second trials of the Super Pop VR game were conducted for children with typical development with two trials for each hand. For children with CP, as many as four trials were conducted for each hand to ensure that at least 6 reaches for each hand were collected. All children were seated on a bench located 2.45 meters in front of the Microsoft Kinect system. The participant's image was projected onto a screen (177.8 cm × 177.8 cm) so it could be viewed clearly by the child. Children were engaged to play a “bubble popping” game with the tested hand. During play, the children were instructed to pop as many yellow bubbles as possible and to pay special attention to popping the green testing bubbles because they were worth double the points of yellow bubbles. Green testing bubbles were located overhead (shoulder abduction at 180 degrees), at shoulder abduction of 135 degrees (active range of motion), and at the side (shoulder abduction of 90 degrees), and they were scaled to the children's arm length, defined as the distance between the shoulder girdle and the wrist crease. To set up the Super Pop VR system for play, children were asked to raise their arm overhead to calibrate the game before playing. In each game, three sets of green testing bubbles appeared at 20 seconds, 40 seconds, and 60 seconds of the game. Prior to data collection, the children participated in a practice trial to make sure the children understood the game rules. The game always started with the children's preferred hand (i.e., less affected hand for children with CP) to help children become familiar with the Super Pop VR game.

Reaching kinematics of the wrist point while touching the three “Super Bubbles” were computed and analyzed using an automated kinematic assessment algorithm. This algorithm creates a baseline model for motor skill assessment by constructing a kinematic chain of links correlated with the dynamics of the human arm.

The following kinematic parameters were computed: path length (the distance the wrist point traveled), movement time (the duration from popping the second bubble to popping the third bubble), the number of movement units (defined as one acceleration and one deceleration phase using von Hofsten's criteria [31]), the average speed of the hand, elbow joint range of motion (ROM), and shoulder joint ROM. The trajectory's path length was computed by calculating and adding the three-dimensional Euclidean distance between each point in the trajectory travelled by the hand. The user's average speed was computed by dividing user's path length by user's movement time. For shoulder and elbow ROM angle calculations, we adhered to the definition of angles between two limb segments used in various biomechanics scenarios; we then calculate the difference between the final joint angle and the initial joint angle [32, 33]. Further details on computing the elbow and shoulder joint angles can be found in Garcia-Vergara et al. [28].

*Bruininks-Oseretsky Test of Motor Proficiency Second Edition (BOT-2)*. To measure changes in arm function after receiving VR intervention, all participants with CP were evaluated with the BOT-2. The BOT-2 assesses proficiency in fine manual control, manual coordination, body coordination, and strength and agility composite. The internal consistency and interrater reliability of BOT-2 are reported to be excellent (0.78–0.97 and >0.92) [34]. A pediatric physical therapist blinded to the study purpose and child's intervention content evaluated the child at home, following the standardized testing procedures listed in the manual.

*Pediatric Motor Activity Log (PMAL)*. PMAL was developed to provide a rating by parents about their child's affected arm on 22 arm-hand real-world functional activities, which are typical for children of 2 to 8 years old (e.g., holding a cup, taking off shoes, and turning a knob). Parents indicated “how often” their child used the more affected hand for each activity on a 6-point scale from 0 (not at all) to 5 (all of the time). Parents also indicated “how well” their child completed these functional activities from 0 (does not use) to 5 (typical for the age). Although PMAL has been used in pediatric constraint-induced movement therapy studies [35, 36], the literature did not report its psychometric properties [37]. Only Lin et al. reported a fair criterion-related validity when comparing the PMAL with WeeFIM and with the Peabody Developmental Motor Scale-2nd Edition. [38]. However, the PMAL was “markedly responsive” to change after intervention [38]. The minimally detectable change (MDC) was 0.67 for the PMAL-Amount (i.e., how often) and 0.66 for the PMAL-Quality (i.e., how well) [38]. The same pediatric physical therapist, blinded to child's intervention status, interviewed the parents and recorded parents' ratings at their home.

### 2.4. Data Analysis

Reaching kinematic parameters of the 3 children with CP were reported as *z* scores, compared with the “normative data” computed from the 11 children with typical development. *z* scores represented the number of standard deviation values by which a given result differed from the mean value for children with typical development. A *z* score value smaller than 1.96 was considered as no difference from children with typical development [39, 40], as a *z* score of 1.96 or less indicated that the score was within 2 standard deviations of the mean of the typically developing children's data. If the change in *z* score between pretest and posttest exceeded 1.65 (*p* = .10), the change was considered statistically significant. We specifically chose 1.65 as our cut-off criterion since we only had three participants and tried to avoid the potential type II error. We also used independent *t*-tests to compare the mean values of reaching kinematics in children with CP and those in children with typical development before, mid, and after intervention. Alpha value was set at 0.10 and SPSS version 18 was used to perform the statistics.

The total point scores on fine motor precision, fine motor integration, and manual dexterity were computed and then converted to a scale score as suggested by the BOT-2 manual [34]. The scale scores then reported as *z* scores, compared with the normative data from BOT-2 manual (mean = 15 and SD = 5) [34]. We used similar criteria to interpret *z* score as we did for reaching kinematic variables. If a *z* score value was smaller than 1.96, it was considered as no difference from the norm values. If the change in *z* score between pretest and posttest exceeded 1.65 (*p* = .10), the change was considered statistically significant.

The change in PMAL score of each child with CP was compared with the MDC reported from the literature: 0.67 for the PMAL-Amount (i.e., how often) and 0.66 for the PMAL-Quality (i.e., how well) [38].

## 3. Results

Among the 3 children with CP, Case  1 and Case  2 were twins with a diagnosis of hypotonic CP, confirmed by their physical therapist. They were born at 32 weeks gestational age and admitted to neonatal intensive care unit for 4 weeks. Case  1 started to walk around 18 months and Case  2 around 22 months. They started receiving outpatient physical, occupational, and speech therapy since they were 3.5 years old. Both children attended home school with flexible hours (4.5 hours per day). Case  3 with a diagnosis of spastic hemiplegic CP was born full term without any known complications. She started to walk around 18 months, which was when her parents noticed her fisted hand and flexed arm. She began receiving outpatient physical and occupational therapy twice per week since she was 2 years old. She attended the public school in her neighborhood. Children with typical development all had right-hand dominance and all had experiences with VR and computer games prior to their participation.

The demographics of the 3 children with CP are shown in Table 1. The kinematic parameters, BOT-2, and PMAL measures for participants are given in Tables 2 and 3. The actual time spent playing VR intervention games per week by each child was 149.5 minutes, 150 minutes, and 62 minutes, respectively.

### 3.1. Reaching Kinematics

All children with and without CP successfully completed the evaluations using the Super Pop VR system in their natural environment (i.e., home setting). The children did not consider the trials as an evaluation. Instead, they thought they played several runs of computer games and enjoyed the testing. Children with CP performed 27 reaches with their more affected arm during the pretest, 33 reaches during the midtest, and 27 reaches during the posttest. Eighty-five percent of the reaches collected were used to compute reaching kinematics, with the rest containing missing data in the trajectories.

In terms of intervention effect, two of the three children with CP (C1 and C3) improved reaching kinematics in path length, movement time, number of movement units, and shoulder joint ROM after the 8-week VR intervention: the *z* score of these kinematic parameters changed more than 1.65 (*p* = .10) between midpoint and pretest or between posttest and pretest (Tables 2(a) and 2(b)). The kinematics in child number 2 with CP also became straighter, faster, and smoother as her path length, movement time, and number of movement units decreased between pretest and posttest. However, her *z* score for these kinematic parameters showed no differences from the values of the typical children. Also, her kinematic parameters in midpoint evaluation was even worse as she was distracted on that day; however, her *z* score also showed no differences from the values of the typical children. Therefore, her change was not considered improved after the VR intervention.

Independent *t*-tests also confirmed the observed trends: at pretest, children with CP had longer path length (*t*(12) = 3.39, *p* = .005), longer movement time (*t*(12) = 4.09, *p* = .002), more number of movement units (MU) (*t*(12) = 3.19, *p* = .01), and smaller shoulder joint ROM (*t*(12) = 2.868, *p* = .01) than children with typical development. There were no differences on elbow joint ROM and average speed of the hand (*p* > .05). At the midpoint test, children with CP still had longer movement time (*t*(12) = 3.05, *p* = .01) and more number of movement units (MU) (*t*(12) = 2.99, *p* = .01) than children with typical development but showed no differences on any of the other kinematic variables (*p* > .05). However, at posttest, none of the variables showed statistically significant differences (*p* > .05).

### 3.2. BOT-2

The total point scores on fine motor precision, fine motor integration, and manual dexterity were computed and then converted to a scale score as suggested by the BOT-2 manual. Only child number 3 with CP changed the *z* score of her manual dexterity from −2.6 to −1.6 between the pre- and postintervention evaluations (Table 3). This showed that her manual dexterity, though still delayed, improved to within 2 standard deviations of the norm values. There were no differences in BOT-2 fine motor scores between pre- and postintervention evaluations for child number 1 and child number 2 with CP.

### 3.3. PMAL

All three children with CP increased their PMAL scores in both “how often” and “how well” categories. The MDC reported from the literature was 0.67 for the PMAL-Amount (i.e., how often) and 0.66 for the PMAL-Quality (i.e., how well). Only child number 3 with CP had a larger change in PMAL between pre- and postintervention evaluation than MDC reported in the literature. That is, only child number 3 with CP improved in the amount and quality of using her affected hand as measured by PMAL.

## 4. Discussion

The results of the present case series demonstrate that the use of Super Pop VR system as a home-based evaluation tool for kinematic metrics is feasible; it was successfully used with all 3 children with CP to collect the desired reaching kinematics in their natural environment. The children enjoyed playing the Super Pop VR game without noticing that their reaching movements were recorded and quantitatively measured; 85% of the reaches performed by the more affected hand could be used to compute reaching kinematics, which is quite compatible with studies using expensive motion analysis system in children and infants (e.g., Chen and Yang [5], Chen et al. [41], and Fetters and Kluzik [42]). Generally, Super Pop VR system was an easy to assemble and implement system. On average it took less than 10 minutes to set up and implement the Super Pop VR system at home and the whole testing duration for 3 rounds of 75 second trials for each hand took around 20 minutes, which gave us 9 reaches for each hand. The children were quite cooperative during evaluation as they considered the whole procedure as part of the computer game playing experience.

The results indicated that 2 of the 3 children with CP showed improvement in the quality of reaching performance after receiving an 8-week VR intervention, as measured by comparing their kinematic performance with typically developing children who provided the “norm” data. Independent *t*-tests confirmed the observations. These results are consistent with our own study [23] as well as Jannink et al.'s [17]. Levac et al. [43] suggested 9 potential active ingredients from VR therapy which could help children with CP improve their motor skills: (1) opportunities for practice, because repetitive task practice advances functional abilities (e.g., children with CP could perform as many as 100 reaches during a 3-minute session when playing the EyeToy game), (2) task specificity between the VR and real-world movements, (3) flexibility to individualize treatment parameters, (4) visual and/or auditory feedback, (5) social play equalization for participating in the play situation, (6) neuroplasticity changes, (7) problem solving through different virtual contexts, (8) motivation since children can select the games they like or compete with peers, and (9) role of a support person (a parent or therapist) with verbal encouragement and feedback. All these components are essential for learning and improving a motor skill. Therefore, these components may be part of the underlying mechanism explaining why VR works.

Interestingly, when using the standardized clinical assessment tools to measure the improvement, only child number 3 with CP improved her manual dexterity of BOT-2 and had parents rating an improvement on “how well” and “how often” she used her affected hand in daily living, as measured by PMAL. However, almost all children with CP improved their reaching kinematics in some aspects, even though only two out of the three children with CP had a change exceeding the cut-off *z* score. Several possible explanations for this finding were proposed. (1) The child with improvement was the only one with relatively impaired arm function and with hemiplegic CP; VR might be of more benefit to train children with hemiplegic CP. Thus, children with mild impairment in arm function and with hypotonic CP might not have a strong benefit in improving their upper limb dexterity using VR intervention. It is also interesting to note that the improvement may not relate to the time spent in practice. Child with hemiplegic CP, on average, practiced only 60 minutes per week, whereas children with hypotonic CP practiced around 150 minutes per week. (2) The VR intervention used in this study had more emphasis on repeated practicing reaching movements in all directions, which was reflected by the improvement in almost every child's reaching kinematics. However, this kind of “specificity of training” in reaching movement might not be able to generalize to other hand-arm functional activities, which were measured in BOT-2 and PMAL. (3) This might be due to the insensitivity of BOT-2 fine motor domain and PMAL to detect minor changes as the therapist narratively described an observation of quality improvement in certain items of BOT-2 but unable to increase the score due to the standardized criterion. Also, the PMAL we used in this study was the original version, which did not have established psychometric properties yet. In our future research, we might need to include the revised PMAL [44], which has established good reliability and validity to detect the minor changes that occur in children with CP.

The study had a few limitations. First, the numbers of children with CP and children with typical development were small with wide variability in the children with CP. Future studies should increase the number of children with and without CP. Second, although inexpensive commercial VR gaming systems increase the accessibility of VR for training children with CP, there were challenges in applying VR systems designed for recreation to do rehabilitation. For example, the level of difficulty of the games and the content of the games (e.g., some violent scenes or age-inappropriate language) might not be suitable for some players. Therefore, future studies may need to evaluate the effects of a tailored VR intervention. Third, our evaluations were limited to pre-, mid-, and posttest. Children's performance might be variable for a number of reasons, such as fatigue and sickness. A better home-based evaluation system should have the ability to evaluate the child's performance in a daily basis. Our Super Pop VR has the potential to serve as a tailored VR intervention as well as a daily evaluation system. Therefore, in future research studies we will use Super Pop VR to conduct a tailored VR intervention program for children with CP and examine children's reaching performance on a daily basis to examine longitudinal improvement in reaching movements in children with CP.

## 5. Conclusion

Our case series show the feasibility of using Super Pop VR as a tool for evaluating reaching kinematics in children with CP. Moreover, the study has shown the improvement of arm function after receiving a VR intervention in a child with hemiplegic CP who had a more impaired arm function, but not in children with hypotonic CP who had relatively good arm function.

## Figures and Tables

**Table 1:** Characteristics of participants in the study.

Participants	Gender	Age in years	Diagnosis	School	Preferred hand	GMFCS/MACS
C1	Female	10	Hypotonic CP	Home school	R	I/II
C2	Female	10	Hypotonic CP	Home school	R	I/II
C3	Female	7	R hemiplegic CP	Public school	L	I/III
Children with typical development	6 females 5 males	8.87 ± 1.87	—	All public school	All R	—

GMFCS: Gross Motor Function Classification System; MACS: Manual Ability Classification System.

**(a):** 

Participant	Path length	Movement time	Movement units	Average speed	Elbow ROM	Shoulder ROM
	(m)	(sec)		(m/sec)	(°)	(°)
C1						
Pretest	0.95	2.41	6.50	0.38	21.53	51.75
Midtest	0.55	1.22	3.78	0.44	15.34	29.94
Posttest	0.63	1.37	4.67	1.02	29.65	23.63
C2						
Pretest	0.44	0.95	2.71	0.72	10.07	36.02
Midtest	0.50	1.27	3.75	0.38	15.05	28.79
Posttest	0.30	0.65	1.88	1.07	9.28	23.55
C3						
Pretest	1.41	3.65	5.29	0.46	32.50	58.43
Midtest	0.51	1.02	5.15	0.57	24.31	52.68
Posttest	0.33	0.88	1.00	0.37	14.87	25.75
Typically developing children	**0.43 ± 0.17**	**0.80 ± 0.26**	**2.23 ± 1.06**	**0.61 ± 0.24**	**16.25 ± 8.88**	**35.49 ± 9.79**
[Lower limit	[0.097	[0.290	[0.152	[0.140	[−1.155	[16.302
upper limit]	0.763]	1.310]	4.308]	1.080]	33.655]	54.678]

The values inside the bracket indicated the upper and lower limit of each reaching kinematic variable in the typically developing children.

**(b):** 

Participant	Path length	Movement time	Movement units	Average speed	Elbow ROM	Shoulder ROM
	(m)	(sec)		(m/sec)	(°)	(°)
C1						
Pretest	2.97	6.09	4.02	−1.09	0.60	1.66
Midtest	0.65^*∗*^	1.57^*∗*^	1.45^*∗*^	−0.82	−0.10	−0.57^*∗*^
Posttest	1.13^*∗*^	2.16^*∗*^	2.29^*∗*^	1.93^*∗*^	1.51	−1.21^*∗*^
C2						
Pretest	0.02	0.55	0.45	0.53	−0.70	0.05
Midtest	0.39	1.79	1.43	−1.08	−0.13	−0.68
Posttest	−0.76	−0.59	−0.34	2.13	−0.78	−1.22
C3						
Pretest	4.44	11.19	2.87	−1.37	0.18	2.08
Midtest	4.16	6.19^*∗*^	2.75	0.57^*∗*^	1.44	−0.18^*∗*^
Posttest	−0.19^*∗*^	0.45^*∗*^	−1.16^*∗*^	−0.83	−0.10	−0.64^*∗*^

*∗* indicates that *z* score changes between intervention and pretest more than 1.65 (*p* = .10).

**Table:** (a) BOT-2

ID	FM precision			FM integration			Dexterity		
	Pre	Mid	Post	Pre	Mid	Post	Pre	Mid	Post
	Scale Scores								
C1	3	2	2	3	3	2	1	2	3
C2	1	2	1	3	3	3	2	3	2
C3	3	1	2	5	1	3	2	1	7

	*z* scores								
C1	−2.4	−2.6	−2.6	−2.4	−2.4	−2.6	−2.8	−2.6	−2.4
C2	−2.8	−2.6	−2.8	−2.4	−2.4	−2.4	−2.6	−2.4	−2.6
C3	−2.4	−2.8	−2.6	−2.0	−2.8	−2.4	−2.6	−2.8	−1.6

**Table:** (b) PMAL

ID	How often			How well		
	Pre	Mid	Post	Pre	Mid	Post
C1	4.636	5.000	5.000	4.591	4.955	4.955
C2	4.773	4.909	4.909	4.682	4.818	4.818
C3	2.045	2.500	2.955^*∗*^	1.955	2.045	3.182^*∗*^

The change score for “how often” needed to exceed 0.67 and for “how well” needed to exceed 0.66 to be clinically meaningful.

*∗* indicates that the PMAL score exceeded MDC between pre- and postintervention evaluation.

## References

[1] Jones M. W., Morgan E., Shelton J. E., Thorogood C. (2007). Cerebral palsy: introduction and diagnosis (Part I). *Journal of Pediatric Health Care*.

[2] Fedrizzi E., Pagliano E., Andreucci E., Oleari G. (2003). Hand function in children with hemiplegic cerebral palsy: prospective follow-up and functional outcome in adolescence. *Developmental Medicine and Child Neurology*.

[3] Krigger K. W. (2006). Cerebral palsy: an overview. *American Family Physician*.

[4] CDC (2015). *Cerebral Palsy*.

[5] Chen Y.-P., Yang T.-F. (2007). Effect of task goals on the reaching patterns of children with cerebral palsy. *Journal of Motor Behavior*.

[6] Boyd R. N., Morris M. E., Graham H. K. (2001). Management of upper limb dysfunction in children with cerebral palsy: a systematic review. *European Journal of Neurology*.

[7] Fetters L. (1991). Measurement and treatment in cerebral palsy: an argument for a new approach. *Physical Therapy*.

[8] Ketelaar M., Vermeer A., Hart H., Van Petegem-van Beek E., Helders P. J. M. (2001). Effects of a functional therapy program on motor abilities of children with cerebral palsy. *Physical Therapy*.

[9] Sakzewski L., Ziviani J., Boyd R. (2009). Systematic review and meta-analysis of therapeutic management of upper-limb dysfunction in children with congenital hemiplegia. *Pediatrics*.

[10] Sakzewski L., Ziviani J., Boyd R. N. (2014). Efficacy of upper limb therapies for unilateral cerebral palsy: a meta-analysis. *Pediatrics*.

[11] novak I., Mcintyre S., Morgan C. (2013). A systematic review of interventions for children with cerebral palsy: state of the evidence. *Developmental Medicine and Child Neurology*.

[12] Löwing K., Bexelius A., Carlberg E. B. (2009). Activity focused and goal directed therapy for children with cerebral palsy: do goals make a difference?. *Disability and Rehabilitation*.

[13] Fetters L. (2010). Perspective on variability in the development of human action. *Physical Therapy*.

[14] Galil A., Carmel S., Lubetzky H., Heiman N. (2001). Compliance with home rehabilitation therapy by parents of children with disabilities in Jews and Bedouin in Israel. *Developmental Medicine and Child Neurology*.

[15] Parsons T. D., Rizzo A. A., Rogers S., York P. (2009). Virtual reality in paediatric rehabilitation: a review. *Developmental Neurorehabilitation*.

[16] Snider L., Majnemer A., Darsaklis V. (2010). Virtual reality as a therapeutic modality for children with cerebral palsy. *Developmental Neurorehabilitation*.

[17] Jannink M. J. A., van der Wilden G. J., Navis D. W., Visser G., Gussinklo J., Ijzerman M. (2008). A low-cost video game applied for training of upper extremity function in children with cerebral palsy: a Pilot Study. *Cyberpsychology and Behavior*.

[18] Reid K., Campbell K. (2006). The use of virtual reality with children with cerebral palsy: a pilot randomized trial. *Therapeutic Recreation Journal*.

[19] Rostami H. R., Arastoo A. A., Nejad S. J., Mahany M. K., Malamiri R. A., Goharpey S. (2012). Effects of modified constraint-induced movement therapy in virtual environment on upper-limb function in children with spastic hemiparetic cerebral palsy: a randomised controlled trial. *NeuroRehabilitation*.

[20] Galvin J., Levac D. (2011). Facilitating clinical decision-making about the use of virtual reality within paediatric motor rehabilitation: describing and classifying virtual reality systems. *Developmental Neurorehabilitation*.

[21] Galvin J., McDonald R., Catroppa C., Anderson V. (2011). Does intervention using virtual reality improve upper limb function in children with neurological impairment: a systematic review of the evidence. *Brain Injury*.

[22] Wang M., Reid D. (2011). Virtual reality in pediatric neurorehabilitation: attention deficit hyperactivity disorder, autism and cerebral palsy. *Neuroepidemiology*.

[23] Chen Y.-P., Kang L.-J., Chuang T.-Y. (2007). Use of virtual reality to improve upper-extremity control in children with cerebral palsy: a single-subject design. *Physical Therapy*.

[24] Chen Y.-P., Lee S.-Y., Howard A. M. (2014). Effect of virtual reality on upper extremity function in children with cerebral palsy: a meta-analysis. *Pediatric Physical Therapy*.

[25] Donovan K., Lord S. E., McNaughton H. K., Weatherall M. (2008). Mobility beyond the clinic: the effect of environment on gait and its measurement in community-ambulant stroke survivors. *Clinical Rehabilitation*.

[26] van den Noort J. C., Ferrari A., Cutti A. G., Becher J. G., Harlaar J. (2013). Gait analysis in children with cerebral palsy via inertial and magnetic sensors. *Medical and Biological Engineering and Computing*.

[27] García-Vergara S., Howard A., Shumaker R. (2014). Three-dimensional fitts law model used to predict movement time in serious games for rehabilitatioin. *Virtual, Augmented and Mixed Reality: Applications of Virtual and Augmented Reality*.

[28] Garcia-Vergara S., Serrano M. M., Chen Y., Howard A. M. Developing a baseline for upper-body motor skill assessment using a robotic kinematic model.

[29] Chen Y.-P., Caldwell M., Dickerhoof E. (2014). Game analysis, validation, and potential application of EyeToy play and play 2 to upper-extremity rehabilitation. *Rehabilitation Research and Practice*.

[30] Klingels K., Jaspers E., Van de Winckel A., De Cock P., Molenaers G., Feys H. (2010). A systematic review of arm activity measures for children with hemiplegic cerebral palsy. *Clinical Rehabilitation*.

[31] von Hofsten C. (1991). Structuring of early reaching movements: a longitudinal study. *Journal of Motor Behavior*.

[32] Norkin C. C., White D. J. (2009). *Measurement of Joint Motion: A Guide to Goniometry*.

[33] Gajdosik R. L., Bohannon R. W. (1987). Clinical measurement of range of motion: review of goniometry emphasizing reliability and validit. *Physical Therapy*.

[34] Bruininks R. H., Bruininks B. D. (2005). *Bruininks-Oseretsky Test of Motor Proficiency Second Edition Manual*.

[35] Case-Smith J., DeLuca S. C., Stevenson R., Ramey S. L. (2012). Multicenter randomized controlled trial of pediatric constraint-induced movement therapy: 6-month follow-up. *American Journal of Occupational Therapy*.

[36] Deluca S. C., Case-Smith J., Stevenson R., Ramey S. L. (2012). Constraint-induced movement therapy (CIMT) for young children with cerebral palsy: effects of therapeutic dosage. *Journal of Pediatric Rehabilitation Medicine*.

[37] Wallen M., Ziviani J. (2013). Caution regarding the Pediatric Motor Activity Log to measure upper limb intervention outcomes for children with unilateral cerebral palsy. *Developmental Medicine and Child Neurology*.

[38] Lin K.-C., Chen H.-F., Chen C.-L. (2012). Validity, responsiveness, minimal detectable change, and minimal clinically important change of the Pediatric Motor Activity Log in children with cerebral palsy. *Research in Developmental Disabilities*.

[39] Cohen J. (1988). *Statistical Power Analysis for the Behavioral Sciences*.

[40] Portney L. G., Watkins M. P. (2008). *Foundations of Clinical Research: Applications to Practice*.

[41] Chen Y.-P., Fetters L., Saltzman E., Holt K. G. (2002). Making the mobile move: constraining task and environment. *Infant Behavior and Development*.

[42] Fetters L., Kluzik J. (1996). The effects of neurodevelopmental treatment versus practice on the reaching of children with spastic cerebral palsy. *Physical Therapy*.

[43] Levac D., Rivard L., Missiuna C. (2012). Defining the active ingredients of interactive computer play interventions for children with neuromotor impairments: a scoping review. *Research in Developmental Disabilities*.

[44] Wallen M., Bundy A., Pot K., Zivian J. (2009). Psychometric properties of the pediatric motor activity log used for children with cerebral palsy. *Developmental Medicine and Child Neurology*.

